# Supplementary material for: Are carbon emissions trading and green financial instruments synergistic? -Comprehensive quantitative research based on content analysis
Source: PLoS One. 2024 Mar 7;19(3):e0298601. doi: 10.1371/journal.pone.0298601 (PMC10919601; doi:10.1371/journal.pone.0298601)
Supplement: S1 Data — (ZIP) [file pone.0298601.s001.zip › Data/地级市Z_F工作报告.zip!/╔╜╢1⁄2/═■║ú╩╨/└┤╫╘╣1⁄2╓┌║┼í╛┬φ┐╦╩2╛▌═°í┐_2010.docx]

各位代表：

现在，我代表威海市人民政府向大会做工作报告，请予审议，并请各位政协委员和其他列席人员提出意见。

一、2009年政府工作的回顾

过去的一年，在省委、省政府和市委的坚强领导下，市政府团结和带领全市人民，坚持以邓小平理论和“三个代表”重要思想为指导，全面贯彻党的十七大和十七届三中、四中全会精神，扎实开展深入学习实践科学发展观活动，紧紧围绕市委确定的工作思路，积极作为、科学务实，坚定信心、共克时艰，努力化挑战为机遇，有效遏制了经济下滑态势，较好地完成了十五届人大三次会议确定的主要目标任务。

――扩内需、保增长取得积极成果。全市生产总值达到1969.4亿元，比上年增长12.8%，比预期目标高2.8个百分点。财政总收入达到217.4亿元，地方财政收入达到102.5亿元，增长9.5%，其中国税增长9.5%、地税增长12.2%。社会消费品零售总额增长19.1%，固定资产投资增长24.2%。

――调结构、转方式取得明显进展。高新技术产业产值占工业总产值的比重提高2.8个百分点，消费占生产总值的比重提高2.1个百分点，税收占地方财政收入的比重提高2.1个百分点，三次产业比重由7.4：61.2：31.4调整为6.9：60.6：32.5。预计万元生产总值能耗降低5.72%，二氧化硫和化学需氧量排放量分别削减2.86%和2.7%。

――惠民生、保稳定取得实际成效。财政用于民生方面的支出比上年增长30.1%。新增城镇就业4.7万人，转移农村劳动力1.3万人，实现高校毕业生就业0.9万人。新增城镇养老、医疗保险参保人员3.7万人和24.4万人，新增新型农村社会养老保险参保人员7.1万人。城镇居民人均可支配收入达到20117元，农民人均纯收入达到9226元，分别增长8.5%和8.6%。

在应对国际金融危机冲击、保持经济平稳较快发展的重大考验面前，我们认真执行中央、省的调控政策和一揽子计划，坚持把抓当前与谋长远、保增长与调结构、扩内需与稳外需、稳发展与促改革、惠民生与保稳定有机地结合起来，统筹兼顾，突出重点，有针对性地采取了一系列措施：

第一，突出了企业扶持。针对危机冲击下企业面临的实际困难，把企业振兴作为保增长的重中之重来抓，组织开展了新一轮联系企业工作，最大限度地减负让利，千方百计地化解融资难题，全方位优化发展环境，帮助企业提升抵御风险的能力。扎实开展了减负让利促三保、优化服务提效能、排忧解难办实事和化解矛盾促和谐四项主题实践活动，取消调整收费项目262项，减轻企业和社会负担1.2亿元，减免缓社会保险1.2亿元，各级落实支持企业发展的财政资金11.5亿元。提供临时还贷扶持资金30.7亿元，为183家企业解决了资金链问题；通过支持担保机构帮助653家中小企业融资26.2亿元。积极争取降低了电价，下调了经营用汽价格，年可减少企业支出1.6亿元。出台了鼓励银行业金融机构发展奖励办法，引进了招商银行等5家金融机构，与省农行、中国信保等金融机构签订了战略合作协议，异地银行授信额度达到176亿元。大力推进了银企对接，新增金融机构贷款217.5亿元，是上年信贷增量的2.1倍。强化了企业市场化融资服务，天润曲轴上市融资8.4亿元，新北洋上市获得核准。骨干企业快速膨胀，中小企业活力不断增强，去年新增销售收入过10亿元的企业16家、过20亿元的4家、过50亿元的1家，三角集团销售收入达到130亿元，威高集团实现利税11亿元；个体私营企业由上年的8.5万户发展到9.6万户。

第二，突出了项目建设。抓住中央实施积极财政政策和适度宽松货币政策的有利机遇，坚持把项目建设作为扩内需、保增长的着力点，强化领导分包和部门责任制，千方百计地上新项目、上高端产业项目。按照“快重准实”的要求，实施中央、省投资项目197个，争取扶持资金17.9亿元；强化了项目的监督检查，前三批项目基本实现“三个百分之百”。围绕重点产业领域，引进过千万美元的外资大项目31个、过亿元的内资大项目63个，其中36个已经开工建设。强化跟踪推进、督查考核、联动服务三个机制，推进了100项市级重点工程，完成投资350.5亿元，占规模以上投资的31.6%；长会口大桥、威海港三四期等45个项目建成并投入使用，荣乌高速公路荣文段、青烟威荣城际铁路、石岛湾核电站等重点工程的前期工作进展顺利，机场三期扩建、威海港国际客运中心搬迁等重点项目加快推进。启动了十大高端产业聚集园区建设，实施过亿元项目38个、过10亿元项目16个，完成投资115.2亿元，主导企业销售收入达到94.5亿元。投资结构进一步优化，服务业、高新技术项目比重分别达到50.6%和15.2%，比上年提高4.8个和0.1个百分点。

第三，突出了自主创新。深入开展了“自主创新年”活动，强化政策引导和资金支持，进一步提高了企业的创新能力。在高层次人才聚集区建设方面，新建了光威国家级国际科技合作基地、全国大学生科学就业与创业示范基地，实施国外引智项目62个，引进各类人才1万多名。在产学研结合密集区建设方面，威高和光威两个国家工程实验室、广泰空港国家级工程技术研究中心、好当家国家级企业技术中心获得正式批准，新增7家省级企业技术中心、5家省级工程技术研究中心，各类省级以上企业技术研发机构达到102家；新建6家院士工作站、3个产业技术创新战略联盟、2个高校产学研战略合作联盟，达成各类产学研合作协议85项。在科技成果转化汇集区建设方面，新建了威海技术交易中心，新认定高新技术企业15家，总数达到78家；取得重要科技成果140项，专利授权量增长20%。

第四，突出了结构调整。坚持把结构调整作为主攻方向，强化战略规划指导，出台了蓝色经济区建设、高端产业聚集区建设、工业调整振兴、旅游文化产业发展等意见和规划，进一步明确了产业发展的方向和重点。海洋经济六大基地建设取得新突破，远洋捕捞、名优养殖、“三品”加工能力进一步增强，渔业经济总产值增长8.3%；造船业逆势快速膨胀，整船制造能力达到200万载重吨，完工量90万载重吨，出口8.5亿美元，分别增长125%、73%和29%；临港产业在整合中得到新发展，港口吞吐量达到4100万吨，增长8%；新能源产业发展步伐加快，新核准风电装机容量34万千瓦。高端产业聚集区建设加速推进，战略性产业集群加力发展，传统优势产业加快升级，三角、威高、光威、华东数控、新北洋等高端产业重点企业发展势头迅猛，新创中国驰名商标4件、各类省级品牌28个、省优质产品生产基地3个，三角集团获得首届省长质量奖。八大产业的集聚效应和支撑作用进一步增强，汽车、医药产业销售收入分别增长36.5%和58.3%。服务业发展水平进一步提升，推进了旅游文化产业整合发展，举办了国际人居节、渔具博览会等34项大型会展活动，服务业增加值增长12.9%，旅游总收入增长18.7%。

第五，突出了改革开放。坚持把改革开放作为保增长、促转型的强大动力，不失时机地推进重要领域和关键环节改革，下大力气强化产业招商，千方百计稳定外需，在困境中实现了招商引资的稳定增长和对外贸易的稳步回升。组建了12个产业招商办公室，组织了23次国内外重点招商活动，合同外资达到5.9亿美元，实际到帐外资5.5亿美元，分别增长3%和4.6%；到位内资324亿元，增长12.8%，项目的规模和质量都有较大提升。出台了7项稳定外需的扶持措施，落实了33亿元出口货物退免税，设立了900万元专项资金强化出口企业扶持和重点市场开拓；在全市范围内广泛深入地开展了农产品质量安全示范区建设，典型经验在全国推广。外贸进出口总额达到106.1亿美元，降幅逐步收窄。强化对企业“走出去”的引导和支持，新核准设立境外企业15家，对外承包劳务营业额增长15%。高技术产业开发区、经济技术开发区、工业新区的承接能力进一步增强，石岛管理区、文登南海新区、乳山海湾新区等园区拉开了发展新框架。深化了国资监管体制改革，推进了国有企业改制重组。成立了政府投融资管理中心，实现政府融资的借、用、管、还一体化。健全了城建重点工程融资机制，推进了财政支出绩效评价和公务卡制度改革。加快推进了“大城管”体制建设，理顺了停车场管理体制，完成了“小公共”退市工作。开展了集体林权和农村土地承包经营权流转制度改革。有步骤地推进了政府机构改革和文化管理体制、医药卫生体制、义务教育学校绩效工资等多项改革。

第六，突出了“三农”工作。围绕城乡统筹发展，全面实施了“十大惠农工程”，着力提高农业生产能力，改善农村生产生活条件，拓宽农民增收渠道，加快推进新农村建设。通过扶持农业龙头企业和农民专业合作社、推广苹果提质增效等新技术，提高了特色种植、特种养殖水平，保障了主要农产品稳定增产。粮食总产达到104.4万吨，增长4.3%；肉、蛋产量分别增长13.9%和12.9%。继续实施了农村道路、自来水改造工程，对310个村庄实施了环境综合整治，完成病险水库除险加固154座，新建镇村污水处理设施14座，新增户用沼气池1.1万座。强化了农村社区综合服务设施建设，新建扩建镇卫生院、村卫生室137处，新建改造农家店412处。落实家电、汽车下乡政策，农民受益7373万元。推进了农村学校“食暖行”工程、农村文化大院、农家书屋和体育健身设施建设。

第七，突出了人居环境建设。坚持建管并重，开展了城市精细化管理行动，加强了生态保护和环境治理，进一步提高了城市规划建设管理水平。加快推进了城市控规和专业性规划的编制工作，加大了对海岸线、山体和城市绿地的保护力度，拆除违法建筑5.3万平方米。市区66个城中村完成整体拆迁，33个村完成整体回迁，新建回迁房317.8万平方米，回迁安置1.4万户。编制了市区村镇布局规划，启动了农村居住工程，新建农村住房4.8万户，改造危房9723户。宝泉广场、环翠楼、金线顶等重点工程顺利实施，城市污水处理、供热供气能力进一步增强。加快了城市精细化管理信息系统建设，集中力量整治了户外广告、“五小行业”、建筑垃圾、工地扬尘、机动车尾气、近岸污水排放等问题。整顿和规范了房地产市场秩序。推进了节能减排重点工程和监测设施建设，城市环境综合整治定量考核位居全省第一。加大了土地违法违规问题的整治力度，关闭市区采石场58处。推进了植树造林和园林绿化，加强了美国白蛾、松材线虫等病虫害防治，成片造林11万亩，被评为“国家森林城市”和“生态省建设先进市”。

第八，突出了保障民生。坚持把富民作为第一导向，出台和落实了一批保障和改善民生的政策措施，努力提升人民群众的幸福感和安全感。开展了创建国家级创业型城市活动，加强了大中专毕业生就业工作，发放小额创业担保贷款7172万元，保持了就业形势基本稳定。强化长效机制建设，在新型农村社会养老保险、城乡生活困难居民救助、在校大学生医疗保险、异地居住退休人员医保、孤残儿童保障、农村社区建设、农村学前教育、敬老院管理服务等方面制定了一批新政策，是我市社会民生政策出台最密集、实施最集中的时期之一。提高了城乡低保、企业离退休人员基本养老金标准，扩大了社会保障和救助范围，将低保边缘家庭纳入了相应救助范畴。市人力资源市场、救助管理站、东发老年公寓等一批民生基础设施投入使用。建设经济适用住房2506套，新增廉租住房946套，改造残疾人危房400户。

第九，突出了社会协调发展。把推动经济发展与社会进步紧密结合起来，以提升公共服务能力为重点，全面推进各项社会事业繁荣发展。实施了“五大文化惠民工程”，加强了10个文化产业示范基地建设，开展了“幸福威海文化年”活动，启动了数字电视整体平移，文登市被评为“山东历史文化名城”。深入推进了素质教育，大力实施中小学校舍安全工程，新建扩建中小学20所，被评为“全国义务教育均衡发展工作先进地区”。强化了社区卫生服务体系建设，建立了联防联控机制，全力应对手足口病和甲型H1N1流感疫情。出台了农村计划生育困难家庭专项救助意见，发放各项计划生育奖扶资金4300万元，惠及22万人。圆满完成了十一届全运会的项目承办和参赛工作，举办了第二届市运会，提高了大型赛事承办能力和体育产业发展水平。加强了民主法制建设，深入开展了普法工作。推进了城乡社区建设，环翠区获得“全国和谐社区示范区”称号。加强了国防动员、人民防空、民兵预备役工作。推进了平安威海建设，加强了社会治安综合治理和社会矛盾纠纷排查调处，健全了应急管理体系，完成了国庆安保任务，各类安全事故下降24.5%。援川和对口帮扶工作进一步加强。民族、宗教、妇女儿童、外事、侨务、对台、史志、档案、地震、气象、仲裁、慈善等事业也取得了新成绩。

第十，突出了政府自身建设。从优化服务环境、转变政府职能入手，加快推进服务型政府建设。结合深入学习实践科学发展观活动，扎实开展了“问政于民、问需于民、问计于民”和“听民声、体民情、促发展”等活动，解决涉及群众切身利益的各类问题1万多个。围绕群众关心的重点问题，组织召开了21次论证会，推进了依法决策和民主决策。加强了行政审批中心建设，推进了相对集中部门行政许可权工作，审批项目集中率由40%提高到71%。强化了政府信息公开、行政复议、行政执法和行政效能监察，实行了纪检监察派驻机构统一管理。狠抓机关工作纪律整顿，认真实施了禁酒令，较好地解决了少数工作人员不遵守作息时间、工作日中午饮酒、酒后驾车等问题。自觉接受人大、政协的监督，认真办理了59件人大代表建议、173件政协委员提案，积极邀请民主党派和无党派人士参政议政。认真履行“一岗双责”，从源头上反腐倡廉。

各位代表！受金融危机和外部环境的影响，全市外贸出口总额下降8.5%，没有完成预期目标。对此，在《政府工作报告》的附件中作出了专门说明。

各位代表！2009年是新世纪以来我国经济发展最为困难的一年。回顾一年来的工作，我们遇到的困难要比预料的多，但取得的成果要比预期的好。成绩的取得，是党中央、国务院和省委、省政府正确领导的结果，是市委总揽全局、掌舵把关和市人大、市政协依法监督、鼎力支持的结果，是全市人民齐心协力、埋头苦干、扎实工作的结果。在此，我代表市人民政府，向全市人民，向各位人大代表和政协委员，向各民主党派、工商联、各人民团体和社会各界人士，向驻威部队指战员、武警官兵和公安干警，向各位老领导、老同志，向所有关心支持威海发展的海内外朋友，致以崇高的敬意和衷心的感谢！

在总结成绩的同时，我们也清醒地看到，我市经济社会发展中还存在一些矛盾和问题，主要表现在：经济回升的基础还不稳定、不巩固、不平衡，保持平稳较快增长的压力还很大；经济结构性矛盾依然突出，服务业比重不高、消费需求拉动力不强、财税结构不优等问题有待进一步解决；自主创新能力还不够强，生态环境保护的压力还比较大；招商引资、稳定外需仍面临较大困难，部分领域的改革仍需加快推进；农民持续增收的困难较多，农村生产生活环境仍需改善；社会事业发展还存在薄弱环节，增加就业、保障困难群众生活、维护和谐稳定的压力依然很大；政府部门的服务意识、行政效能和廉政建设都有待进一步加强，等等。对此，我们一定保持清醒的头脑，采取有力措施，认真加以解决。

二、2010年政府工作的目标要求

各位代表！2010年是实施“十一五”规划的最后一年。做好今年的政府工作，对战胜当前的危机和困难、为“十二五”发展打好基础具有十分重要的意义。2010年政府工作的总体要求是：坚持以科学发展观为指导，全面贯彻党的十七大和十七届三中、四中全会精神，牢牢把握积极作为、科学务实的工作基调，以建设蓝色经济区、高端产业聚集区为总抓手，更加注重提高经济增长质量和效益，更加注重经济发展方式转变和经济结构调整，更加注重推进自主创新和改革开放，更加注重改善民生和保持社会和谐稳定，努力实现经济平稳较快发展，全面完成“十一五”规划，为“十二五”规划的实施打下良好基础，在建设创新开放宜居幸福的现代化新威海征程上迈出新的步伐。

主要预期目标为：生产总值增长10%，地方财政收入增长11%，万元生产总值能耗降低4.27%，化学需氧量和二氧化硫排放量分别削减1.5%和1%，固定资产投资增长20%，社会消费品零售总额增长17%，外商直接投资与上年持平，实际利用内资增长10%，外贸进出口增长5%，城镇居民人均可支配收入增长8%，农民人均纯收入增长8%，城镇登记失业率控制在2%以内，人口自然增长率控制在1‰以内。

各位代表！上述预期目标的确定，充分考虑到今年经济形势的复杂性、严峻性和多变性，考虑到我们的发展基础、条件和实际需要，并与中央、省的预期目标进行了衔接，既积极稳妥，又留有余地。在实际工作中，我们将积极谋求更好更快的发展。在促增长、稳发展的同时，将把更多的精力放到调结构、转方式上来，放到惠民生、促和谐上来，放到抓创新、求突破上来，努力使政府的工作更加符合形势和发展的要求，符合人民群众的期待。在今年的工作中，我们将突出以下四个重点：

一是千方百计巩固回升向好势头。紧紧围绕保持经济平稳较快发展，毫不动摇地贯彻执行中央和省出台的一系列政策措施，毫不放松地加强项目建设和企业扶持，毫不懈怠地抓好扩大内需、稳定外需的各项工作，进一步夯实经济发展基础，增强经济增长的稳定性。

二是坚定不移推进经济转型升级。紧紧抓住调结构、转方式的核心要求，在加快建设蓝色经济区和高端产业聚集区中促进产业升级，在大力强化自主创新和节能减排中转变发展方式，在着力提升质量和增创品牌中增强发展优势，在积极推进城镇化和新农村建设中拓展发展空间，进一步增强结构调整的主动性，努力在发展中促转变、在转变中谋发展。

三是集中力量增强体制机制活力。紧紧抓住经济社会发展中的矛盾和问题，坚持用改革的方法破解发展难题，以开放的思路增强发展活力，进一步解放思想、转变观念，不断消除体制机制障碍，增强发展的内在动力，提升应对危机和困难的能力，形成区域竞争的新优势，为在新一轮经济发展中抢占先机创造条件。

四是全力以赴增进社会民生福祉。紧紧抓住保障和改善民生这一根本，把发展经济与改善民生结合起来，把为群众解难题、办实事放在更加重要的位置，把促进社会稳定和谐作为发展的保障，全力维护人民群众的利益，满足人民群众的需求，进一步增强发展的协调性，努力做到发展为了人民、发展依靠人民、发展成果由人民共享。

各位代表！今年仍是机遇与挑战并存、困难和希望同在的一年。不管困难有多大，压力有多重，我们都将牢记政府的职责，以如履薄冰的危机感、时不我待的紧迫感、守土有责的使命感，脚踏实地，努力工作，充分调动方方面面的积极性，千方百计破难题，凝心聚力谋发展，攻坚克难，乘势而上，不断开创科学发展的新局面！

三、2010年政府工作的重点

（一）推进蓝色经济区和高端产业聚集区建设，在强化自主创新中加快经济结构调整和发展方式转变。牢固树立“一盘棋”思想，认真落实省委、省政府建设山东半岛蓝色经济区和胶东半岛高端产业聚集区的战略部署，进一步强化规划指导，加强与周边地区对接，充分发挥自身比较优势，努力争当先行区。

蓝色经济区，继续加大六大基地建设力度，加快构筑海洋特色鲜明、区域布局合理、产业素质优良、生态环境良好、带动能力较强的蓝色经济体系。全年海洋产业增加值增长15%，占生产总值的比重提高1个百分点。海产品生产加工基地，注重巩固优势，做优做强远洋捕捞、名优养殖和海产品深加工，实施好海洋生物引种育种工程，抓紧建立远洋渔业综合基地，新增海洋牧场10万亩，海产品生产加工产值增长8%。船舶修造基地，注重提升能力，以核心技术研发和配套企业发展为突破口，支持三大造船聚集区、十大重点造船企业快速发展，力争年内整船制造能力达到240万载重吨。港口物流基地，注重上档升级，实施“兴港强市”战略，进一步优化港口布局，做好威海港新港区资源整合工作，加快推进威海港国际客运中心、南海新港、乳山港一期、石岛新港扩建等工程建设，大力发展五大临港工业区，做好码头、泊位对外开放工作，优化口岸“大通关”环境，力争全年港口吞吐量达到4400万吨。滨海旅游休闲度假基地，注重突出特色，努力打造半岛滨海高端旅游示范区和国内知名滨海旅游目的地。新能源和现代石化基地，注重战略合作，着力构建全方位、多元化的能源体系，编制风力发电中长期规划，做好镆铘岛石化项目的对接工作。按照科学开发海洋资源的要求，大力推进海洋科技创新体系建设，加快海洋技术成果转化；实施好海洋生态环境保护工程，加大海岸线、滨海湿地保护力度，推动海洋资源的有序开发和海洋经济的可持续发展。

高端产业聚集区，按照“新兴产业抓集群、传统产业创品牌”的要求，认真落实八大产业调整振兴规划，力争全年高端产业增加值占生产总值的比重达到38%以上。在新兴产业培植上，依托优势龙头企业，强化政策支持和要素保障，集中力量扶持培育十大高端产业聚集园区，抓好三角集团全钢胎、威高集团植入医疗器械和心内耗材、华东数控核电锻铸件、光威集团碳纤维生产线、新北洋特种打印扫描设备、新船重工和黄海造船整船研发及零部件配套等项目，年内园区主导企业新增销售收入100亿元以上，力争用3-5年打造10个销售收入过百亿的高端产业聚集园区。以高端、高质、高效为方向，着力建设好石岛湾核电站及风电、光伏发电等新能源项目，培植好镀膜玻璃、骨科材料、铝镁合金等新材料项目，实施好华夏、迪沙、达因等新医药项目，抓好太阳能电池、新型元器件、智能电子、软件开发等新信息项目，打造产业发展的新亮点、新优势。在传统产业扶持上，以地毯、家纺、渔具、皮具、工具、电机、服装、木工机械等行业为重点，强化品牌的提升带动作用，鼓励企业从生产加工型向品牌导向型转变，从争创品牌向经营品牌转变，把传统优势产业基地打造成国内核心聚集区。大力实施“名牌兴企、质量兴市”战略，引导农业、工业和服务业的各类企业加强品牌和标准化建设，支持优势行业共同打造专业商标品牌基地、地理标志证明商标、集体商标等区域品牌，争取新增中国驰名商标2件、山东著名商标8件、山东名牌产品6个。大力发展电子商务和基于电子商务的物流产业，创新市场营销模式。加快用高新技术提升传统产业的步伐，支持骨干企业大力推进技术改造，加快产品升级换代，争取150个项目列入省级以上技术创新项目，工业技改投资增长16%。

自主创新是经济结构调整和发展方式转变的基础和动力，是蓝色经济区和高端产业聚集区建设率先突破的关键。坚持把自主创新作为第一动力，认真开展好第三个“自主创新年”活动，以提高企业创新能力为重点，加快推进高层次人才聚集区、产学研结合密集区、科技成果转化汇集区建设。促进“引资”与“引智”相结合，加强国内外人才引进工作，争取年内新增市级以上引智成果示范推广基地10家，组织实施引智项目60个，引进各类人才1万名。引导和扶持企业强化创新平台建设，争取新增市级以上工程技术研究中心10家、企业技术中心6家、省级以上重点实验室2家、省级以上产品质量检验检测中心2家。充分调动企业的积极性，发挥驻地高校的作用，深入推进产学研合作，共建中国工程物理研究院技术中心威海中心，争取再成立1-2个产业技术创新战略联盟，签订产学研合作协议50项。大力实施“千项技术创新工程”，争取发明专利申请量增长10%，取得重要科技成果80项，高新技术产业产值占工业总产值的比重达到36%。

（二）继续强化项目建设，以发展服务业为重点努力扩大消费需求。把项目建设作为第一抓手，充分发挥投资推动经济增长、调整经济结构的先导作用，集中力量推进重点项目建设，保持合理的投资规模。年内投资283.6亿元，加快实施100个市级重点项目，主要包括青烟威荣城际铁路、荣乌高速公路荣文段、烟海高速公路乳山段、机场三期扩建、江家寨立交桥、市区内环快速路、统一路南延等18个基础设施项目，威高工业园三期、三角工业园、西霞口造船、东安黑豹扩建、航海食品等32个现代制造业项目，广泰空港、东生能源等23个高新技术项目，华东重工、史丹利工具、大世汽车等13个外资项目，市粮食仓储物流中心、苏宁电器等14个服务业和社会事业项目。继续强化领导分包和部门责任制，完善跟踪服务和督查机制，协调推进项目建设。进一步激活民间投资，鼓励民间资本进入基础产业、基础设施、市政公用事业、社会事业等领域。积极做好中央、省资金的争取和使用工作，确保达到“三个百分之百”的要求。

全面落实刺激消费的各项政策，增强消费对经济增长的拉动力。落实工资指导线和最低工资制度，提高中低收入群体的消费能力。扩大社会保障覆盖面，增强群众消费预期。做好家电、汽车摩托车下乡工作，落实农机购置补贴、家电和汽车以旧换新等政策。发展社区商业、物业、家政等便民服务业，扩大旅游休闲、文化娱乐、教育培训、体育健身等服务型消费，推动消费结构优化升级。继续实施“万村千乡”和“农超对接”工程，加强农村现代流通服务网络建设。严厉打击扰乱市场秩序、假冒伪劣、虚假广告、商业欺诈等各种违法犯罪行为，维护消费者合法权益。

服务业是扩大消费的重要增长点，是结构调整的重要着力点。要强化旅游文化产业的龙头地位，落实进一步加快旅游业发展的意见，扎实开展旅游文化产业发展行动。进一步理顺旅游管理体制，整合旅游资源，加快推进华夏城、大乳山二期、仙姑顶、汤泊温泉、金海游艇俱乐部、好当家滨海旅游度假区等重点项目，开展旅游服务质量提升年活动，深度开拓客源市场，争取全年旅游总收入增长15%。落实文化产业振兴规划，大力培育以数字、网络等高新技术为支撑的新兴文化业态，加快发展文化创意、影视服务、出版发行业，支持神游华夏、梦海等文化演艺产业快速发展。突出发展生产性服务业，出台鼓励生产性服务业发展的意见，扶持金融保险、现代物流、信息服务、科技服务、商务服务和法律服务等产业发展，争取民生银行等股份制商业银行在我市设立分支机构，加快打造一批开拓能力强的服务外包企业。大力发展会议、节庆经济，培育特色会展品牌，促进相关产业发展。大力整顿房地产市场秩序，促进房地产业健康发展。

（三）以产业招商为重点提升开放层次和水平，促进外需稳定增长。把产业招商作为推进产业优化升级的有效途径，坚持“走出去”与“引进来”相结合，进一步强化部门责任，完善考核机制，组织好日韩、港台、欧美等10次境外招商活动和北京、上海、深圳等10次合作对接会，举办好5个专业洽谈会、两次跨国公司招商会和一系列重点客商“威海行”活动，争取在重点产业领域再引进一批大项目。坚持以我为主、择优选资，大力实施基于产业链整合的招商模式，引导外资重点投向战略性新兴产业、高新技术产业、高端制造研发环节和现代服务业，重点抓好造船、汽车、新材料、新能源等12个产业链条的规划和招商。

加快转变外贸发展方式，以保市场、保份额、促平衡为重点，千方百计稳定外需。实施“以质取胜”战略，调整优化进出口结构，扩大机电、高新技术和自主知识产权产品出口。深入推进农产品质量安全示范区建设，搞好新加坡食品基地、美国肉食鸡养殖基地、韩国食品企业采购物流中心等项目合作，进一步打响农产品出口品牌。推动加工贸易转型升级。深入实施市场多元化战略，利用好市场开拓基金和出口信誉保险机制，加大中东、中亚、南美、东欧等新兴经济体和发展中国家市场开拓力度，举办国际渔具博览会、中日韩绿色食品展等大型展会。

深入推进企业战略重组和区域对接合作。加强与跨国公司驻中国机构、国内大型企业的联络，推动我市骨干企业与国内外强企合作，再引进一批有实力的战略合作伙伴。强化外商投资服务，鼓励在威投资企业增资扩股。加强与产业大市和资源大省的对接，推动优势资本和优质资源的合作。支持各类有条件的企业“走出去”发展，开展境外资源、技术合作开发，开拓境外工程市场和高端劳务市场。搭建对接合作平台，主动融入黄河三角洲高效生态经济区建设。

（四）扎实推进重要领域和关键环节改革，进一步完善有利于科学发展的体制机制。一是深化国有企业改革。进一步理顺市属国有企业国资监管体制，加快推进公司制改造，健全法人治理结构和业绩考核、分配调控机制，确保国有资产保值增值。二是理顺支持中小企业发展的体制机制。扎实开展保护中小企业发展行动，建立健全灵活的机制以促进科技创新、中小企业成长、特色产业提升和小企业培育四个计划的实施。扩大中小企业发展专项资金规模，完善中小企业融资、技术改造、产业聚集、社会化服务等方面的政策服务体系，为中小企业发展提供有力保障。三是进一步创新融资机制。大力推进银企对接，健全续贷过桥机制，不断优化金融生态环境，争取全年新增贷款15%以上。加强各类融资平台和担保机构建设，设立市创业投资公司，扩大小额贷款公司、村镇银行试点。加快新北洋、泓淋电子、华鹏玻璃、三角轮胎、俚岛海洋科技等企业的上市进程，全力争取高区“新三板”试点。四是完善财税管理体制。探索增收节支和经营财政的新机制，健全资源有偿使用制度，大力培植财源，拓展增收渠道。创新税源管理机制，完善纳税服务、信息管税和内控监督体系。强化部门预算管理，全面推开公务卡制度，健全财政支出绩效评价机制，大力压缩公用经费和一般性支出。做好省直管县财政体制改革试点工作。五是稳妥推进农村各项改革。进一步完善农村土地流转机制，加强土地流转服务中心建设。建立农田水利等农村基础设施建设、管护、运营机制。全面推开集体林权制度改革。六是健全价格调控机制。完善价格应急监测预警机制，强化民生价格监管，做好清费治乱减负工作，发挥价格调节基金的作用，保持重点商品价格水平的基本稳定。七是深化医药卫生体制改革。坚持统筹兼顾，远近结合，抓好基本医疗保障制度、国家基本药物制度、基层医疗卫生服务体系、基本公共卫生服务均等化和公立医院改革试点等五项重点改革。建立政府主导下的药品配送机制，鼓励医药流通企业兼并重组，打造有竞争力的医药流通体系。八是扎实推进政府机构改革。全面完成市县级政府机构改革任务，稳妥推进事业单位绩效工资制度改革和岗位设置管理。抓好文登市和张村镇两个省级综合配套改革试点。

（五）夯实“三农”发展基础，扎实推进新农村建设。进一步落实强农惠农政策措施，增加涉农补贴规模，促进农业增产、农民增收、农村发展，扩大内需增长空间。

深化农业结构调整，切实增强农业综合生产能力。进一步加大农业科技和良种推广力度，扩大新兴特色产品基地面积；全面实施苹果提质扩量增效工程，建设示范基地30处；抓好畜牧业的规模化养殖、标准化生产，加强动物疫病防治，确保粮食、肉、蛋、奶和果品产量稳定增长。大力扶持农业龙头企业和农民专业合作社，密切利益衔接机制，进一步提高农业产业化水平。积极推进新型农产品经营服务体系建设。加快发展无公害产品、绿色食品、有机食品和地理标志产品，提升农产品总体质量和档次。

加强农村基础设施建设，进一步改善农村生产生活条件。完成6座中型和105座小型病险水库除险加固工程，全面消除病险水库。新增农业节水灌溉面积6万亩。加快推进农村居住工程，年内新建农房3.4万户、改造危房5446户。再对200个村集中开展农村环境综合整治。推进农村公路、自来水改造和沼气建设，改造120个村的自来水工程，新建沼气工程32处、户用沼气池5000座。加强农村社区综合服务中心建设，新建农家店100处。

增强农村公共服务能力，提高农村社会保障水平。全面推开新型农村社会养老保险，进一步完善农民养老保障体系，扩大社会化保险覆盖面。继续完善农村三级医疗卫生服务网络，改建10处镇卫生院和183个村卫生室。加强农村敬老院的规范化管理和对口帮扶，对农村贫困、大病老年人进行专项救助。继续推进农村学校“食暖行”工程，改善农村办学条件。全面普及镇综合文化站和农村文化大院，进一步活跃农村文化生活。

（六）大力推进新型城镇化，提升城镇规划建设管理水平。遵循城镇化发展的规律，顺应产业聚集、园区开发和城市拓展的趋势，考虑人口、资源、环境等可持续发展的因素，扎实开展新型城镇化建设行动，形成新的增长极、增长带、增长面。

搞好园区和小城镇建设，带动城市有序拓展。按照集约开发、产业集聚、突出特色的原则，进一步突出高区、经区的龙头作用，继续举全市之力支持工业新区建设，引导9个省级以上开发区和各类园区优化空间布局，强化产业集聚效应，完善社会服务功能。集中力量抓好4个国家重点镇、10个省级中心镇和12个市级中心镇建设，增强小城镇的发展活力，培育县域经济的增长点。

坚持“提升内涵、控制外延”，加快推进精品城市建设。做好新一轮小城镇总体规划、村镇建设规划和停车场、供排水等专项规划，继续整治私搭乱建，依法维护规划的严肃性和权威性。中心市区实施6大系列、23项重点工程，推进双岛湾休闲旅游度假城、工业新城、临港产业服务区、五渚河流域生态休闲度假区“两城两区”建设，抓好宝泉广场、金线顶、塔山公园等工程；荣成、文登、乳山抓好110项城建重点工程。加快推进市社会福利院、文登社会福利中心、环翠区体育中心等项目，进一步提高公共服务水平和保障能力。

抓好城中村改造和城市环境综合整治。以回迁安置为重点，全力做好城中村改造工作，完成4个村663户的拆迁扫尾，建设8490户、79.9万平方米的回迁安置房，搞好小区的配套设施，切实兑现承诺。加强对环海路、世昌大道、青岛路、环山路、文化路两侧的综合整治，实施农副产品批发市场、水产品批发市场、西城路农贸市场搬迁改造，推进光亮工程改造和城市园林绿化，进一步改善城市面貌。

坚持建管并重，深入开展城市精细化管理行动。整合部门职能，建立联动高效的“大城管”体制。加快城市管理信息系统建设，推进首期70平方公里区域的精细化管理。积极实施公交优先战略，继续整顿规范城市交通秩序和停车秩序。进一步规范户外广告，加强建筑工地垃圾清运和噪声管理，加大“五小行业”整治力度。推进“菜单式”物业管理收费改革。加强城乡社区建设，扩大农村社区试点，加快市区村改居步伐，积极推进低保救助、就业指导、养老服务、医疗卫生、纠纷调处、社会治安、文体活动等社会事务的社区管理服务一体化。

（七）强化节能减排发展低碳经济，进一步加大环境保护力度。鼓励发展低碳经济和循环经济，积极开发和使用绿色清洁能源，提高资源循环利用和废弃物综合利用水平。严格执行环境影响评价制度，落实投资项目节能评估和审查制度，加快淘汰落后产能，强化对100户重点用能企业的节能监察，抓好80户重点排污企业的视频监控系统建设，完成3家电厂脱硫工程。推进建筑节能和供热计量改造。鼓励中水回用和再生水利用。大力倡导绿色消费和节约理念，进一步推动节约型社会建设。

全面强化环境保护和污染治理。建立空气质量分区公告制度，对机动车尾气、建筑工地扬尘、工业废气、秸秆燃烧等主要污染源进行持续治理。加快建设威海二污、文登南海、乳山银滩等7处污水处理厂和18处镇污水处理设施，羊亭、初村污水处理设施投入运行，生活污水集中处理率达到90%以上。加强饮用水水源地保护，抓好威海湾3条入海暗河的排污整治，继续推进母猪河、乳山河等重点流域治理。加快实施市垃圾处理厂二期工程，推进农村生活垃圾集中处理。认真落实新一轮土地利用总体规划，稳妥处理闲置土地。加强矿产资源管理，规范地下水及地热资源的开采使用，对市区88处采石场实施综合治理。继续抓好“五林”工程，做好松材线虫、美国白蛾等有害生物防治和森林防火工作，全年造林8.6万亩。扎实推进生态市创建工作，争取文登、乳山生态市创建通过国家考核验收。

（八）坚持统筹兼顾，大力发展社会事业。进一步推进城乡教育均衡发展，深化素质教育，提高中小学的教育质量和办学水平。大力发展职业教育，开展好学前教育先进县和示范镇创建工作。组织实施好中小学校舍安全工程，年内新建改造校舍61万平方米。继续加强与山大、哈工大等高校的共建。进一步完善公共文化服务体系，繁荣广播电视、新闻出版事业，加强文化市场管理，继续开展好“幸福威海文化年”活动。强化投入保障，落实好全民健身条例和体育强市建设意见，办好国际长距离铁人三项赛、国际休闲帆船锦标赛等品牌赛事，做好22届省运会备战和参赛工作。积极争创全国文明城市，深入开展群众性精神文明创建活动，加强未成年人思想道德建设，强化社会信用体系建设。完善计划生育利益导向机制，稳定低生育水平，提高出生人口素质。做好史志编修工作，加强民生档案资源管理。抓好双拥共建和优抚安置工作，加强国防教育、国防动员、民兵预备役和人民防空建设。进一步做好民族、宗教、妇女儿童、外事、侨务、对台、气象等各项工作。继续加强援川和对口帮扶工作，确保完成援建任务。精心组织好“十二五”规划的编制工作。

（九）着力保障和改善民生，千方百计维护社会稳定。一是全力做好就业工作。深化国家级创业型城市创建工作，鼓励和支持全民创业。抓好大中专毕业生就业工作，加快大学生创业就业基地建设。继续做好援企稳岗工作，强化职业技能培训和公共就业服务，加强对困难群体的就业援助。全年新增城镇就业3.5万人，转移农村劳动力1万人。二是进一步完善社会保障体系。采取灵活政策扩大各类社会保险的覆盖面，继续提高企业离退休人员基本养老金标准。扎实做好养老保险省级统筹，稳步推进医疗、失业、工伤、生育保险市级统筹，城镇职工养老、工伤保险参保人数分别增加1.1万人和1.6万人。三是加大社会救助力度。提高最低生活保障水平，城市居民由每月330元提高到345元，农村居民由每年1200元提高到1400元，城乡低保覆盖率分别达到1%和3.2%。加强困难群众的医疗、教育、临时救助等专项救助工作。做好红十字会和慈善工作。加强残疾人的康复、保障工作，帮扶1000户农村贫困残疾人，免费为1000名白内障患者实施复明手术。四是提高公共卫生服务水平。强化政府责任和投入，进一步提高人均公共卫生服务经费标准，基本医疗保险参保覆盖率由80%提高到85%，新型农村合作医疗财政补助标准由每人每年90元提高到110元。积极探索社区卫生服务发展的新路子，推进社区卫生服务机构标准化建设。全面实施好健康教育、免疫接种、妇幼保健、职业病和慢性病管理等公共卫生服务项目，加强手足口病、甲型H1N1流感等传染病的防控。五是加强保障性住房建设。实施经济适用住房货币化补贴政策，对符合条件的购房家庭每户补贴6万元；将人均建筑面积小于15平方米的住房困难家庭全部纳入住房保障范围。

全面推进“平安威海”建设，切实维护社会稳定大局。加强民主法制建设，搞好“五五”普法工作。深入开展“安全生产基层基础年”活动，实施重点领域安全专项整治，坚决遏制重特大事故发生。进一步理顺管理体制，强化食品药品质量安全监管。抓好防洪、抗震、消防、疾控等设施的优化升级，建立健全应急安保体系，强化应急演练，切实提高交通安全、环境安全等突发事件的应急处置能力。发挥信访、人民调解、仲裁、法律援助机构的作用，强化职能部门的责任，完善联动机制，共同帮助群众维权，及时化解医患纠纷、劳资纠纷、质量纠纷及各类社会矛盾。坚持重心下移，警力下沉，加强社会治安综合治理，依法严厉打击各类违法犯罪活动，切实增强群众的安全感。

四、切实加强政府自身建设

各位代表！新的形势下，人民群众对政府工作的期待和要求越来越高，我们肩负的责任和担子也越来越重，唯有不断地提升素质、优化服务，才能不辱使命、不负重托。

（一）强化宗旨意识。巩固深入学习实践科学发展观活动成果，强化宗旨意识教育，牢固树立尊重人民、敬畏人民、感谢人民、服务人民、依靠人民的理念，全心全意为人民群众解难题、办实事。建立健全“三问”长效机制，开展好“向市民承诺、为市民办事、请市民监督”活动，进一步畅通行风热线、12345市长公开电话、市长信箱、社情民意调查中心等联系渠道，充分利用网络的互动作用，听民声、体民情、集民智，切实提高工作的针对性和有效性。在机关事业单位广泛开展服务品牌创建活动，进一步优化行政服务环境。坚持学以致用，加强公务员培训和普法教育，进一步增强服务本领和能力。

（二）坚持依法行政。进一步健全重大行政决策听证、合法性论证、实施情况反馈和决策责任追究制度，推进政府依法决策、科学决策和民主决策。结合政府机构改革，进一步健全部门间的沟通配合机制，深化相对集中行政处罚权工作。全面落实行政执法责任制，规范行政处罚自由裁量权，严肃查处失职不作为、越权乱作为。组织开展规范行政执法活动，努力提高基层执法能力，构建规范、公正、和谐的执法环境。加强收费管理，规范收费行为，实行收费项目动态清理，切实减轻企业和社会的负担。

（三）提高行政效能。加强行政审批中心建设，完善电子审批和电子监察系统，进一步提高集中办事程度，简化办事程序，为人民群众提供更多便利。进一步严肃政纪，严格执行禁酒令、婚宴和车改纪律等规定。坚持求真务实，坚决反对形式主义和虚报浮夸，深入基层开展调查研究，切实转变工作作风。健全快捷高效的工作落实机制，加强重大决策事项督查，完善目标绩效管理考核，把科学发展的着力点放在真抓实干上，在狠抓落实中更好地为发展服务、为基层服务、为群众服务。

（四）自觉接受监督。依法接受人大及其常委会的监督，主动接受政协的民主监督，认真办好人大代表建议和政协委员提案。大力推行政务公开，深入开展民主评议政风行风活动，自觉接受新闻舆论和社会公众监督。加大执法监察、效能监察和廉政监察的力度，加强对重点领域和项目的审计。进一步推进政府诚信建设，做到立言为信、立行必果、有诺必践。

（五）搞好廉政建设。认真落实党风廉政建设责任制，着力推进惩治和预防腐败体系和制度建设，做到用制度管权、按制度办事、靠制度管人。抓好群众反映的热点难点问题的专项治理，加快查处教育医疗、征地拆迁、涉农利益等方面的突出问题，切实纠正部门和行业不正之风。继续加大案件查办力度，严肃查处各类违纪违法案件，以反腐倡廉的实际成效取信于民。

各位代表！做好今年的各项工作，任务艰巨，责任重大。让我们更加紧密地团结在以胡锦涛同志为总书记的党中央周围，高举邓小平理论和“三个代表”重要思想伟大旗帜，全面落实科学发展观，在市委的坚强领导下，充分调动一切积极因素，凝聚全市人民的智慧和力量，奋发进取，开拓创新，扎实工作，为建设创新开放宜居幸福的现代化新威海做出新的更大贡献！
